# Relapsed refractory multiple myeloma with CNS involvement successfully treated with Elranatamab: first reported case

**DOI:** 10.3389/fimmu.2023.1276295

**Published:** 2023-10-13

**Authors:** Yasa Gul Mutlu, Sureyya Yıgıt Kaya, Senem Maral, Elif Melek, Zafer Baslar, Leylagul Kaynar, Omur Gokmen Sevindik

**Affiliations:** ^1^ Department of Hematology, Istanbul Medipol University, Istanbul, Türkiye; ^2^ Department of Hematology, Istanbul University-Cerrahpasa, Istanbul, Türkiye

**Keywords:** Elranatamab, multiple myeloma, bispecific Ab, BCMA, CNS involvement

## Abstract

Central nervous system (CNS) involvement in multiple myeloma (MM) is a rare and challenging complication associated with poor prognosis and limited treatment options. Emerging T-cell directing therapies, such as bispecific antibodies (bsAbs) and chimeric antigen receptor T cells (CAR-T), have shown remarkable success in treating MM, but their efficacy in CNS involvement remains unclear. Elranatamab, a humanized bispecific antibody targeting B-cell maturation antigen (BCMA) and CD3-expressing T cells, has demonstrated promising results in relapsed refractory MM. However, its efficacy in treating CNS-MM has not been reported. We present a case of a 37-year-old male MM patient with CNS involvement who has been successfully treated with Elranatamab.

## Introduction

Emerging T-cell directing therapies, such as bispecific antibodies (bsAbs) and chimeric antigen receptor T cells (CAR-T), have demonstrated remarkable responses and outcomes in extensively treated and treatment-resistant patients (1). Despite significant advances in the treatment of MM, central nervous system (CNS) involvement remains a challenging and rare complication that can lead to severe neurological symptoms and impact patient outcomes. Elranatamab is a humanized bispecific antibody that targets both B-cell maturation antigen (BCMA)-expressing multiple myeloma (MM) cells and CD3-expressing T cells (2). Elranatamab has shown a promising overall response rate of 70% in heavily pretreated myeloma patients. However, there is currently limited or no available data regarding its use and efficacy specifically for CNS involvement in multiple myeloma. Here we report early and effective use of Elranatamab for relapsed refractory Multiple Myeloma patient with CNS involvement.

## Case description

**Figure 1:**
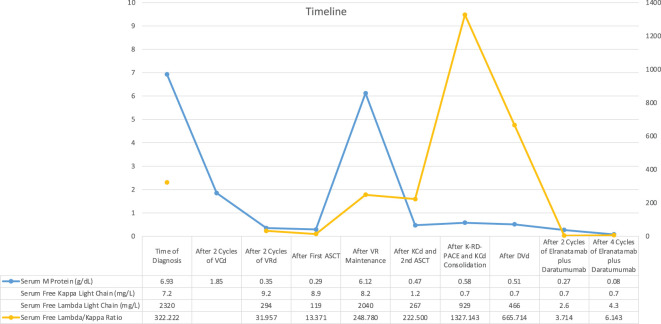
Timeline of patient treatment schema.

**Figure 2:**
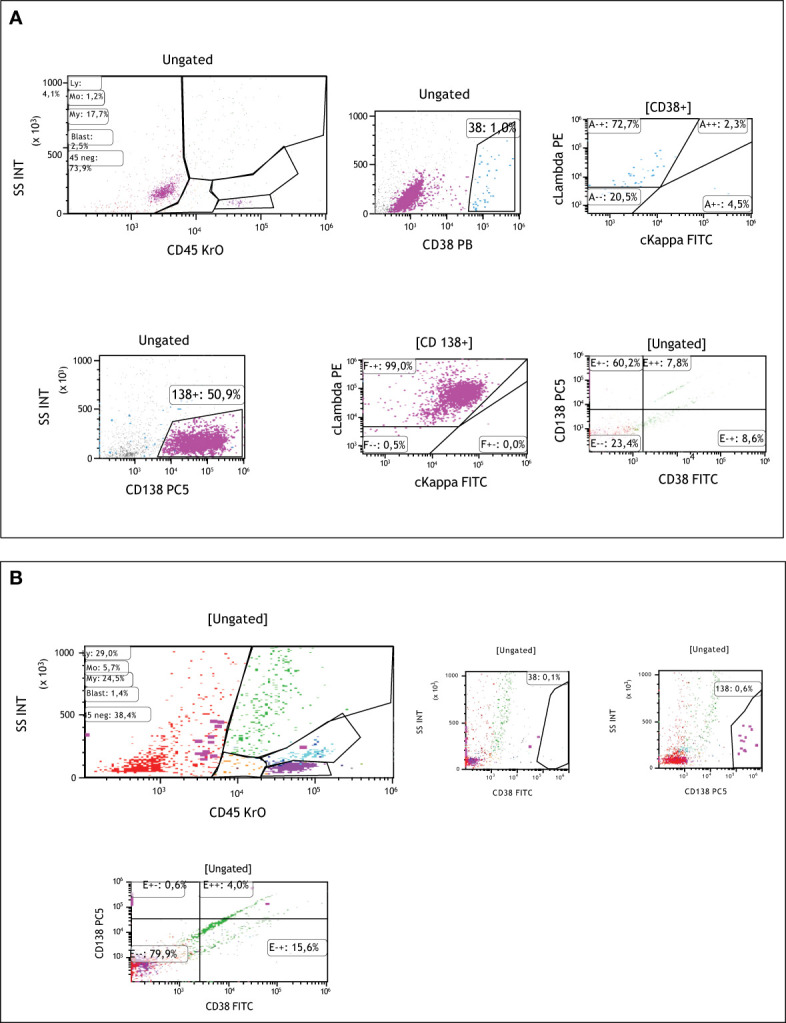
**(A)** Flowcytometric analyses of CNS fluid at the time of diagnosis showing CD138 positive lambda clonal plasma cells and decreased CD38 positivity after Daratumumab therapy. **(B)** Flowcytometric analyses of CNS fluid after 2 cycles of Elranatamab showing decreased CD138 positive lambda clonal plasma cells.

**Figure 3:**
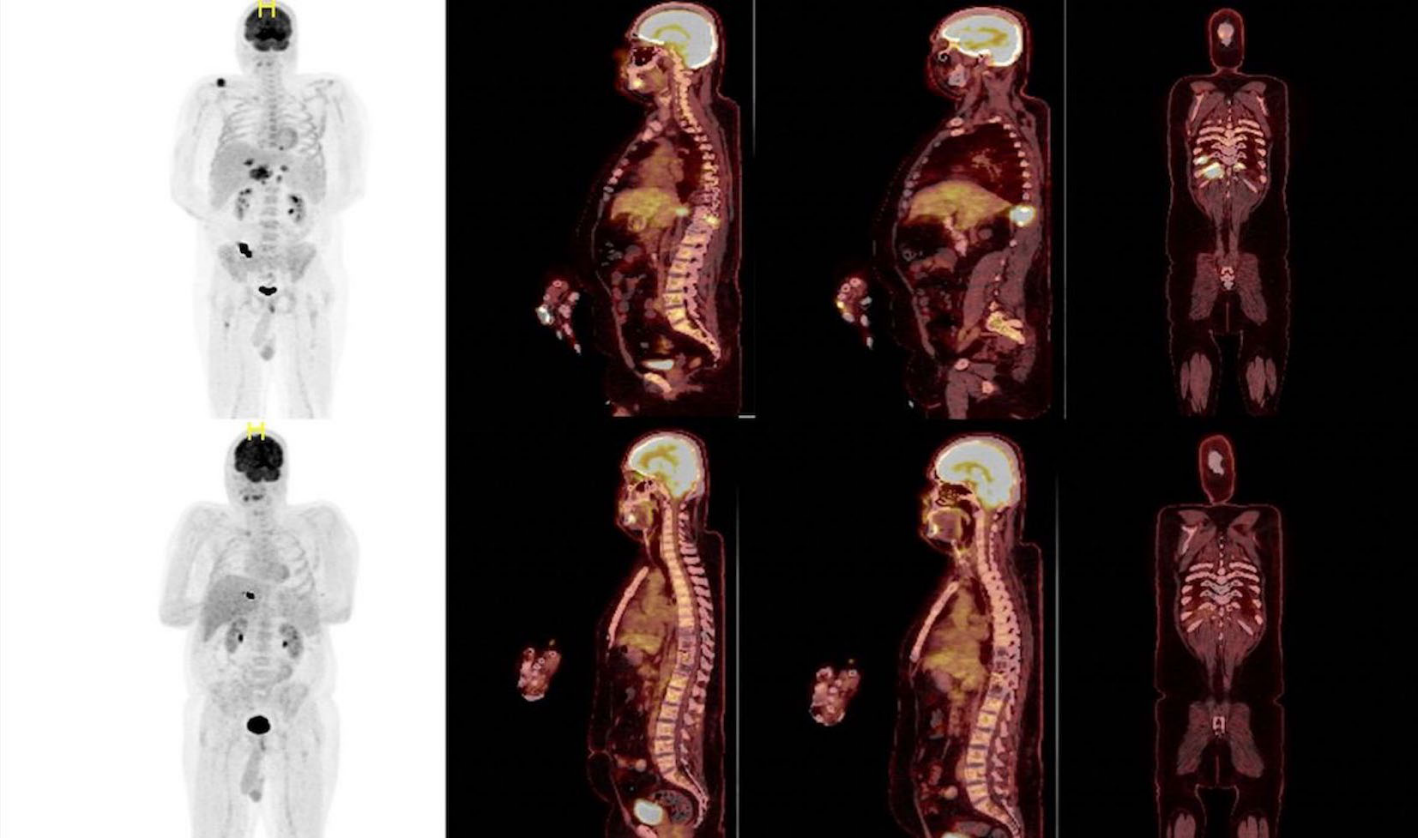
PET CT images are showing a complete remission before (on the top) and after (bottom) Elranatamab therapy.

A 37-year-old male patient presented with a sudden onset back pain and fatigue. Laboratory test results revealed normochromic normocytic anemia, elevated total protein, and an increased serum creatinine level indicating renal failure. The diagnosis of IgG lambda Multiple Myeloma ISS III and R-ISS II was confirmed with an increased number of plasma cells in the bone marrow aspiration and biopsy. Cytogenetic analysis showed 46 XY karyotype with no additional myeloma specific molecular abnormalities including 17 p deletion, translocation t(11,14), t(14,16), t(4,14) and amp/gain 1q. 30 gene next generation sequences analyses of bone marrow at the time of diagnosis showed KRAS mutation with VAF (variant allele frequency) 37.2% and CALR mutation 48% VAF. PET CT (Positron Emission tomography) scan showed multiple lytic lesions and spinal bone-derived plasmacytomas. Bortezomib, Cyclophosphamide, Dexamethasone (VCD) was initiated as a first-line treatment, and after the normalization of renal functions, the patient proceeded with Bortezomib, Lenalidomide and Dexamethasone (VRD). Very good partial response (VGPR) response according to M protein level was reached after 4 cycles of induction therapy however, PET CT scan showed persistent Fluorodeoxyglucose (FDG) avid solitary lesion paravertebrally located in the spinal cord. 3-Gray (Gy) involved-field radiation therapy (IFRT) was applied before preceding to transplantation. Although maintenance therapy initiated after the stem cell transplantation because of the high-risk features such as extramedullary nature of the disease, patient was in VGPR only 6 months. Due to increased number of extramedullary lesions and increased FDG uptake while on maintenance therapy Carfilzomib, Cyclophosphamide and Dexamethasone (KCd) was started and after 2 cycles of therapy patient underwent second autologous transplantation due to prolonged cytopenia. Cytopenia was resolved after transplantation but no improvement observed regarding to disease. Patient was on KCd as a consolidation therapy after the transplant. PET-CT, which was obtained in three months after transplantation revealed increased lytic lesions and bone derived plasmacytomas. Due to extra medullary predominant nature of the disease, Proteasome inhibitor combined chemotherapy regimen, Carfilzomib plus RD-PACE initiated. After two cycles of therapy patient progressed with new plasmacytomas. Daratumumab, Bortezomib, Dexamethasone combination therapy started. After 3 cycles of therapy PET-CT revealed progressive bone lesions. Patient presented with newly onset diplopia, headache, and eye movement abnormalities. Diagnostic lumbar puncture and cranial Magnetic resonance imaging (MRI) were performed in order to exclude Multiple Myeloma involvement in CNS. Cranial MRI did not show any myeloma related cranial lesions or leptomeningeal findings but flow-cytometric analyses of cerebrospinal fluid showed increased clonal CD138 positive plasma cells. CNS involvement was confirmed and weekly Elranatamab 76 mg subcutaneous started by compassionate use of the drug. Treatment schema and response assessment of the patient are detailed in **Figure 1**. Daratumumab continued as scheduled two weeks apart and Dexamethasone given 20 mg weekly. Grade 2 cytokine release syndrome (CRS) fever with low flow oxygen need were required at day 3. One dose of Tocilizumab therapy initiated. No other adverse events observed including ICAN (Immune Effector Cellular Therapy Associated Neurotoxicity), after two cycles of Elranatamab, CNS findings showed significantly increased clonal plasma cells (**Figure 2**). Neurological symptoms regressed. PET CT scan showed complete remission after 4 cycles of therapy (**Figure 3**). Patient is still in remission and has been following up since April 2023.

## Discussion

Soft-tissue plasmacytomas indicate an aggressive form of MM, characterized by autonomous growth of a clone and/or sub clone independent of the bone marrow microenvironment. This condition is associated with high-risk genetic features, increased proliferation, resistance to apoptosis, and treatment resistance (3, 4). Although CNS involvement is extremely rare, the prognosis is even more dismal than extramedullary disease (EMD) in other locations, particularly with leptomeningeal involvement. A multicenter retrospective cohort study investigating CNS involvement in Multiple Myeloma reported a median overall survival of 7 months from the time of CNS involvement (5). In this study, untreated and treated patients had median OS of 2 and 8 months, respectively. While there is no standard of care treatment for CNS involvement in MM, systemic treatment alone or in combination with either intrathecal chemotherapy or radiotherapy has shown a significant improvement in survival when compared to no systemic treatment (5).

Optimal therapy for CNS involvement in MM is not very well established due to small numbers of reported patients with CNS involvement and heterogeneity of their treatments. Traditional approaches such as IT chemotherapy and radiation therapy can lead to dismal survival of 1-2 months (6). The efficacy of new drugs in CNS involvement has been documented, but their full potential remains uncertain. Clinical studies for CNS-MM treatment are scarce, making it a challenging area to address. One of the reasons for this difficulty is the presence of the blood-brain barrier (BBB), which acts as a natural defense, restricting the entry of numerous drugs into the central nervous system (7). The dilemma is whether the BBB is intact and acts as a barrier for drugs but when increased vascular permeability with in the tumor happens it causes transferability or some molecules are able to cross the intact BBB (8). There is limited data on MM agents’ transferability to CSF and their effectivity. A literature review on the cerebrospinal fluid (CSF) transferability of drugs for MM showed that IMIDs and Daratumumab can cross the BBB (9–11). No data are available about other monoclonal antibodies such as Isatuximab and Elotuzumab (12). Previous trials have demonstrated that BCMA CAR-T cells are associated with manageable toxicity and remarkable effectiveness in patients with relapsed/refractory multiple myeloma (R/R MM) (13). However, their potential and suitability for CNS MM treatment have not been determined yet. Yiyun et al. reported 4 CNS MM cases who had been treated with BCMA CART cell therapy and they identified the presence of BCMA CAR-T cells in CSF, and found that BCMA CAR-T cells are safe and effective in treating CNS MM, but the duration of remission may demand improvement (14). Elranatamab is one of the promising bispecific antibodies and has been shown promising results in the setting of relapsed refractory MM, however its effectiveness in CNS involvement remains unclear.

## Conclusion

Elranatamab is one of the promising bispecific antibodies and has been shown promising results in the setting of relapsed refractory MM, however its effectiveness in CNS involvement remains unclear. This is the first CNS-MM case who had been treated with Elranatamab successfully.

## Data availability statement

The raw data supporting the conclusions of this article will be made available by the authors, without undue reservation.

## Ethics statement

The patients provided a written informed consent to participate in this study. Written informed consent was obtained from the patient for the publication of any potentially identifiable images or data included in this article. Written informed consent was obtained from the participant/patient(s) for the publication of this case report.

## Author contributions

YM: Writing – original draft. SY: Writing – review & editing. SM: Writing – review & editing. EM: Writing – review & editing. ZB: Writing – review & editing. LK: Writing – review & editing. OS: Writing – review & editing, Supervision.
